# Development of Biocompatible and Functional Polymeric Nanoparticles for Site-Specific Delivery of Radionuclides

**DOI:** 10.3389/fmed.2015.00063

**Published:** 2015-09-04

**Authors:** Nicolas Lepareur, Loleh Leal E Costa, Maëva Bocqué, Clément Blondelle, Clément Ruello, Marie Desjulets, Nicolas Noiret, Sandrine Cammas-Marion

**Affiliations:** ^1^Comprehensive Cancer Centre Eugene Marquis, Rennes, France; ^2^INSERM UMR-S 991, Rennes, France; ^3^Ecole Nationale Supérieure de Chimie de Rennes (ENSCR), Rennes, France; ^4^UMR 6226 CNRS, Institute of Chemical Sciences of Rennes, Rennes, France

**Keywords:** functional nanoparticles, site-specific targeting, degradable poly(benzyl malate) derivatives, radionuclide encapsulation, technetium-99m

## Abstract

**Introduction:**

Encapsulation of biologically active molecules into nanoparticles (NPs), for site-specific delivery, is a fast growing area. These NPs must be biocompatible, non-toxic, and able to release their load in a controlled way. We have developed a series of NPs based on (bio)degradable and biocompatible poly(malic acid) derivatives, poly(benzyl malate) (PMLABe), with its PEG-grafted stealth analog and target-specific biotin-PEG-*b*-PMLABe one. A lipophilic radiotracer has then been encapsulated into these NPs.

**Methods:**

Monomers were synthesized from dl-aspartic acid. PEG_42_-*b*-PMLABe_73_ and Biot-PEG_66_-*b*-PMLABe_73_ block copolymers were obtained by anionic ring-opening polymerization of benzyl malolactonate in presence of α-methoxy-ω-carboxy-PEG_42_ and α-biotin-ω-carboxy-PEG_66_ as initiators. NPs were prepared by nanoprecipitation. Size, polydispersity, and zeta potential were measured by dynamic light scattering (DLS) and zetametry. ^99m^Tc-SSS was prepared as previously described. Encapsulation efficacy was assessed by varying different parameters, such as encapsulation with preformed NPs or during their formation, influence of the solvent, and of the method to prepare the NPs. After decay, ^99m^Tc-loaded NPs were also analyzed by DLS and zetametry. NPs’ morphology was assessed by transmission electron microscopy.

**Results:**

^99m^Tc-SSS was added during nanoprecipitation, using two different methods, to ensure good encapsulation. Radiolabeled NPs present increased diameters, with identical low polydispersity indexes and negative zeta potentials in comparison to non-radiolabeled NPs.

**Conclusion:**

A radiotracer was successfully encapsulated, but some further optimization is still needed. The next step will be to modify these radiolabeled NPs with a hepatotrope peptide, and to replace ^99m^Tc with ^188^Re for therapy. Our team is also working on drugs’ encapsulation and grafting of a fluorescent probe. Combining these modalities is of interest for combined chemo-/radiotherapy, bimodal imaging, and/or theranostic approach.

## Introduction

Nanomedicine, the use of nanoparticles (NPs) in medicine, is an ever-growing research field as illustrated by the huge amount of scientific publications and the important number of formulations registered for clinical trials, especially for cancer therapies (1). Such a growing interest results from the urgent need for highly efficient anti-cancer drug delivery systems allowing an increased efficacy of the drugs together with a decrease of their non-specific distribution and toxicity (2–4). Indeed, despite better knowledge and understanding of cancer biology, significant improvements in cancer treatments are still needed, specifically to address a large amount of drugs at the site of action, to decrease their toxic side effects and to simplify the administration protocols. In this context, nanoparticulate systems (liposomes, micelles, polymer-based NPs, etc.) have attracted the interest of many researchers and numerous systems have been developed (1). However, the prepared NPs have to answer to very strict specifications: (i) they have to be non-toxic and biocompatible at any stage of their life, until their complete excretion from the body; (ii) they need to have stealth properties and reduced immunogenicity together with ability to carry large amount of drug and to deliver it at a specific site in a sustainable way. Even if undeniable progresses have been achieved in the design of the ideal drug carriers as evidenced by the Food and Drug Administration (FDA) approval obtained by some nanovectors and by the number of NPs in preclinical or clinical phases (1, 5, 6), several properties, such as biocompatibility, drug loading capacity and site-specificity of drug release, need to be further improved. Among the nanoplatforms designed to address these objectives, polymer-based NPs have attracted much interest mainly because of the versatile characteristics of polymers allowing adjustment of the physico-chemical and biological properties of the corresponding nanocarriers. Consequently, several families of polymers have been developed to formulate multifunctional drug carriers with properties adjusted to the considered application (2). However, when designing a polymer family, one must keep in mind that the resulting materials are prepared to be used *in vivo* and that they have to respect the very strict specifications defined above. Another property which can be of importance is the (bio)degradability of the polymers constituting the drug delivery systems, allowing their elimination thanks to the (bio)degradation of the copolymers constituting the NPs, thus avoiding NPs accumulation in the body. However, this means that the degradation process must lead to non-immunogenic, biocompatible, and/or (bio)assimilable low molecular weight molecules that can be eliminated from the body. In this context, several (bio)degradable polymers have been developed and formulated under various nanoplatforms with more or less success (7, 8).

Therefore, the design of (bio)degradable polymers, whose physico-chemical and biological properties can be adjusted either by copolymerization, chemical modifications, or both, is of great importance (9, 10). Poly(β-malic acid) (PMLA) is a very good candidate due to the presence of carboxylic acid pendant groups, which can be chemically modified to introduce molecules of interest, and ester cleavable bounds within the polymer backbone, allowing the degradation of the polymer main chain into biocompatible metabolites (11–13).

Recently, we have synthesized and characterized two amphiphilic derivatives of PMLA presenting stealth properties, the poly(ethylene glycol)-*b*-poly(benzyl malate) (PEG_42_-*b*-PMLABe_73_), and targeting properties, the biotin-PEG_66_-*b*-PMLABe_73_. These block copolymers are able to self-assemble in aqueous media, leading to well-defined NPs designed for site-specific sustainable drug release in the frame of hepatocellular carcinoma (HCC). We showed that an anti-cancer drug model, the Doxorubicin (Dox), can be encapsulated into both stealth and targeted NPs with quite high encapsulation efficiency (14, 15) and that both kind of empty NPs have no significant toxicity against normal and cancer cell lines even at high concentrations (14–16).

Among molecules of interest to be encapsulated, one can include radioisotopes, either for imaging (diagnostic) or therapy, or even both (theranostics). There is an abundant, and ever-growing literature on the subject (17–23). All types of NPs have been radiolabeled (24), from micelles (25), polymeric NPs (26), liposomes (27), nanocapsules (28), or dendrimers (29), to inorganic ones (30). Most of the applications have been in cancer imaging and/or therapy (17), but some applications in cardiovascular (20, 23) and inflammation/infection (31, 32) can be found. Radiolabeling NPs enables the following of the NPs *in vivo* (33), particularly as an image-guided system for targeted delivery (34, 35), or for the controlled delivery of a therapeutic radionuclide (36).

Due to its favorable physical and chemical properties, such as its 6-h half-life, its low energy gamma emission of 140 keV, and relatively low isotope cost, generator-produced technetium-99m (^99m^Tc) has been the most used radionuclide in routine nuclear medicine and is still the subject of numerous investigations, in the search for new radiopharmaceuticals. Logically, attempts to label NPs with ^99m^Tc have been done (26, 30, 37). ^99m^Tc has also the advantage of being part of a matched pair with rhenium-186/188, two isotopes that could be used as therapeutic radionuclides. Rhenium-188 (^188^Re), which is also conveniently generator-produced, like ^99m^Tc, has great potential for radionuclide therapy (38, 39). Moreover, due to similar chemistries, it can be easily replaced with rhenium-188 (E_βmax_ = 2.1 MeV, E_γ_ = 155 keV (15%), t_1/2_ = 17 h) for therapy purposes (40–42).

Because of the growing interest in the site-specific and controlled delivery of radionuclides, we investigated the possibilities to entrap a lipophilic radiotracer, ^99m^Tc-SSS, inside NPs based on PEG_42_-*b*-PMLABe_73_ or Biot-PEG_66_-*b*-PMLABe_73_. Several methods were used to encapsulate ^99m^Tc-SSS with more or less success. The corresponding radiolabeled NPs were characterized in terms of encapsulation efficiency, diameters, polydispersities, zeta potentials, and morphology.

## Materials and Methods

### Materials

Nuclear magnetic resonance spectra (^1^H NMR) were recorded at 400 MHz on a Brucker ARX400 instrument (Billerica, MA, USA). Data are reported as follows: chemical shift (multiplicity, number of hydrogen). The chemical shifts (δ) are reported as parts per million (ppm) referenced to the appropriate residual solvent peak. Abbreviations are as follows: *s* (singlet), *d* (doublet), *t* (triplet), *q* (quartet), dd (doublet of doublet), *m* (multiplet).

Weight average molecular weight (Mw) and molecular weight distribution (Mw/Mn = Ip) values were determined by size exclusion chromatography (SEC) in THF at 40°C (flow rate = 1.0 mL⋅min^−1^) on a GPC 2502 Viscotek apparatus (Malvern Instruments Ltd, Malvern, UK) equipped with a refractive index detector, a LT5000L mixed medium org 300 mm × 7.8 mm gel column and a GPC/SEC OmniSEC software. The polymer samples were dissolved in THF (2 mg⋅mL^−1^). All elution curves were calibrated with poly(styrene) standards.

The dynamic light scattering (DLS) measurements were recorded on a Delsa™ Nano Beckman Coulter apparatus (Fullerton, CA, USA) at 25°C in water. The obtained correlation curve allows calculating the diffusion coefficient (*D*) using the CONTIN method. The hydrodynamic radius (Rh), and therefore the hydrodynamic diameter (Dh), is then calculated from this diffusion coefficient *D* using the Stokes–Einstein equation where k is the Boltzmann constant, T the temperature, and η the medium viscosity (fixed at 0.8878 cP): *D* = kT/6πηRh.

The zeta potential was measured by zetametry using a Delsa™ Nano Beckman Coulter apparatus (Fullerton, CA, USA) at 25°C in water. Laser Doppler electrophoresis in a phase mode was conducted with sequential fast and slow field reversal applying a potential of ±150 V. The measured electrophoretic mobility (μ) was then converted to zeta potential using the Smoluchowski approximation.

Transmission electron microscopy (TEM) was realized using a Jeol 2100 LAB6 microscope (Peabody, MA, USA) equipped with a Gatan Orius 200D camera (Pleasanton, CA, USA). UV spectra were recorded on a Secomam apparatus (Alès, France) at 485 nm. Activity measurements were done in a CRC-127R well-counter (Capintec Inc., Ramsey NJ, USA).

Radiochemical purity (RCP) of the complex was determined using a Perkin-Elmer Cyclone Storage Phosphor Imager (Waltham, MA, USA), with the Packard Optiquant v05.00 software. All chemicals were used as received. Anhydrous THF was obtained by distillation over sodium/benzophenone under N_2_ atmosphere.

### Synthesis of the monomers and polymers

The monomer, benzyl malolactonate (MLABe), was synthesized from dl-aspartic acid according to the previously reported synthesis (43). PEG_42_-*b*-PMLABe_73_ and Biot-PEG_66_-*b*-PMLABe_73_ block copolymers were obtained by anionic ring opening polymerization of MLABe in presence of, respectively, α-methoxy ω-carboxy poly(ethylene glycol), PEG_42_-CO_2_H, and α-biotin ω-carboxy poly(ethylene glycol), Biot-PEG_66_-CO_2_H, as initiators following a protocol described elsewhere (14).

### PEG_42_-*b*-PMLABe_73_

^1^H NMR (400.1 MHz; (CD_3_)_2_CO), δ (ppm): 2.77–2.83 (*m*, 2nH, CO_2_CH_2_C_6_H_5_), 3.56–3.59 (*m*, 4mH (*m* = 42), CH_3_O(CH_2_CH_2_O)_42_), 4.97–5.06 (*m*, 2nH, CHCH_2_CO_2_), 5.39–5.44 (*m*, 1nH, CHCH_2_CO_2_), 7.19–7.27 (*m*, 5nH, CO_2_CH_2_C_6_H_5_). M_NMR_ = 15 860 g/mol for the PMLABe block. SEC (THF, polystyrene standards, 1 mL/min): Mw = 5 430 g/mol, Ip = 1.38.

### Biot-PEG_66_-*b*-PMLABe_73_

^1^H NMR (400.1 MHz; (CD_3_)_2_CO), δ (ppm): 2.83 (*s*, 2nH, CO_2_CH_2_C_6_H_5_), 3.56–3.59 (*m*, 4mH (*m* = 66), Biot(CH_2_CH_2_O)_62_), 5.00–5.05 (*m*, 2nH, CHCH_2_CO_2_), 5.41–5.44 (*m*, 1nH, CHCH_2_CO_2_), 7.19–7.23 (*m*, 5nH, CO_2_CH_2_C_6_H_5_). M_NMR_ = 14 630 g/mol for the PMLABe block. SEC (THF, polystyrene standards, 1 mL/min): Mw = 5 510 g/mol, Ip = 1.45.

### Preparation of the NPs

Nanoparticles were prepared by the nanoprecipitation technique described previously (14). Briefly, a solution of 5 mg of copolymer in acetone was rapidly added to 2 mL of water under vigorous stirring. After 10 min of stirring at room temperature, acetone was eliminated under vacuum.

A slightly modified nanoprecipitation procedure (Gel filtration method) was also used: 5 mg of block copolymer were dissolved in 100 μL of DMF or acetone and 50 μL of DMF or acetone, containing or not the ^185/187^Re[PhCS_3_)_2_(PhCS_2_)] (^185/187^Re-SSS) prepared as previously described (40), were added. The DMF or acetone solution was rapidly added into 1 mL of water under vigorous stirring. This solution was stirred at room temperature for 1 h, passed through a gel filtration PD-10 Sephadex G-25 Medium column (GE Healthcare, Little Chalfont, UK) and eluted with 1.5 mL + 3.5 mL of water.

### Characterization of the nanoparticles

The size (average Dh, polydispersity (Đ) and zeta potential of the formulations were measured by respectively DLS and zetametry, without any dilution of the samples.

Nanoparticles were observed by TEM. NPs’ suspensions in water were placed on a formvar–carbon film coated on a copper grid (300 mesh). After 6 min of deposition at room temperature, non-adherent NPs were eliminated and the sample was stained by phosphotungstic acid (0.1%) for 30 s. TEM imaging was performed under 80 kV accelerating voltage.

### Synthesis of ^99m^Tc-SSS complex

^99m^Tc-SSS complex preparation was adapted from previously described method (44). Briefly, kits containing sodium gluconate and tin (II) chloride were first prepared as described thereafter: 825 mg of sodium gluconate were dissolved in 9.9 mL of distilled water. This solution (0.9 mL) was placed into 10 flasks. In parallel, 8.25 mg of tin chloride (SnCl_2_.2H_2_O) were dissolved into 1.1 mL of 0.1N HCl solution. Then, 0.1 mL of this solution was added in each flask containing the gluconate solution. The flasks were lyophilized and crimped.

One milliliter of [^99m^Tc]-pertechnetate (750–800 MBq), eluted from a ^99^Mo/^99m^Tc generator (ELU III, IBA Molecular, Gif-sur-Yvette, France) was added to a lyophilized kit containing sodium gluconate (75 mg) and SnCl_2_,2H_2_O (0.75 mg) prepared as described above. The mixture was shaken at room temperature for 15 min. Sodium dithiobenzoate (10 mg dissolved into 0.5 mL of 0.9% saline solution) was added, and the solution was heated at 100°C for 15 min. A thin layer chromatography (TLC) was realized in a mixture of hexane and dichloromethane 6/4 as eluent to assess RCP of the complex (R_f_ = 0.62).

### Radiolabeling of the NPs

The ^99m^Tc-SSS complex previously prepared was extracted with 1 mL of dichloromethane. The organic solvent was evaporated at 40°C. Two procedures were tested for radiolabeling of the NPs according to the ones tested for empty NPs and ^185/187^Re-SSS loaded NPs.

#### Standard Nanoprecipitation

Acetone or ethanol (0.5 mL) was then added to the residue to solubilize the complex, and a solution of 5 mg of the selected block copolymers, into 0.5 mL of acetone, was added. The mixture was nanoprecipitated into 2 mL of 0.9% saline solution. After stirring, the organic solvent was eliminated by heating at 45°C, under slight vacuum, for 15 min. The flask was then centrifuged at 3,500 G for 20 min (Awel MF 20-R, Blain, France). The supernatant was discarded and the pellet containing the radiolabeled NPs was resuspended into 2 mL of physiological serum. The radioactivity of the flask before and after centrifugation and resuspension was measured and the encapsulation efficiency was calculated as the ratio of the resuspended pellet activity on total activity before centrifugation.

#### Gel Filtration

DMF or acetone (0.15 mL) was then added to the dried complex. Five milligrams of the selected block copolymer were dissolved into 100 μL of DMF or acetone and 50 μL of the solution containing the ^99m^Tc-SSS complex were added. This organic solution was then rapidly added into 1 mL of water. The mixture was stirred at room temperature for 15 min, passed through a PD-10 Sephadex G-25 Medium column and eluted with 1.5 mL + 3.5 mL of water as described above. The radioactivity of the flask before and after passage on the column was measured and the encapsulation efficiency was calculated as the ratio of the collected fraction’s activity on total activity before filtration.

After total decrease of the radioactivity, ^99m^Tc-radiolabeled NPs were analyzed by DLS to determine their diameter, polydispersity index, and zeta potential. The radiolabeled NPs were also observed by TEM after complete radioactive decay following the procedure described just above. An energy dispersive X-ray spectrometry (EDS) analysis of the radiolabeled NPs was also realized during the TEM measurements.

## Results and Discussion

We have been recently investigating the possibilities of radiolabeling PMLA derivatives-based NPs with the final goal of using such nanocarriers for the dual site-specific carrying of a radioelement (^99m^Tc/^188^Re-SSS) and an anti-cancer drug. To reach this objective, we studied the possibilities to use degradable stealth and/or targeted NPs, constituted by PMLA amphiphilic derivatives, for radionuclides’ encapsulation.

**Figure 1:**
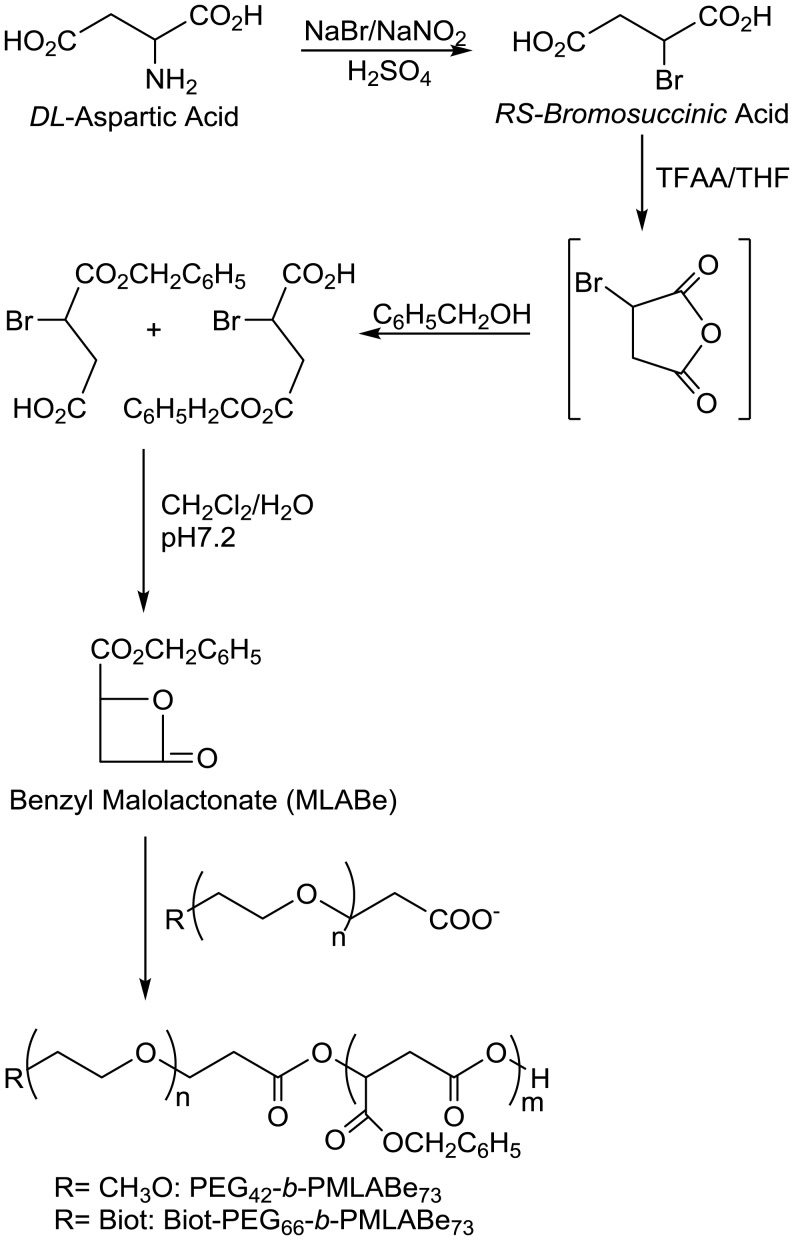
**Synthetic route to PEG_42_-*b*-PMLABe_73_ and Biot-PEG_66_-*b*-PMLABe_73_**.

In a first step, we synthesized two amphiphilic PMLA derivatives, PEG_42_-*b*-PMLABe_73_ and Biot-PEG_66_-*b*-PMLABe_73_, by anionic ring opening polymerization of the MLABe with, respectively, α-methoxy,ω-carboxylate PEG_42_ and α-biotin,ω-carboxylate PEG_66_ as initiator. MLABe was synthesized in four steps, starting from aspartic acid, following a well-described and reproducible synthetic route (Figure 1) (14–16, 43). After purification by precipitation, allowing the elimination of unreacted initiator and low molecular weight oligomers, block copolymers were characterized by ^1^H NMR (structure and molecular weight of the PMLABe block) and by SEC (average molecular weight and polymolecularity).

Polymers’ characteristics are summarized in Table 1. For both block copolymers, the molecular weights of the PMLABe block were calculated from their ^1^H NMR spectra on the basis of the integration of peaks corresponding to the PEG block, whose molecular weight is given by the supplier, and on the integration of peaks corresponding to the PMLABe block. Calculated molecular weights were very similar to theoretical ones chosen at 15,000 g/mol and fixed by the monomer/initiator ratio. On the other hand, the average molecular weights measured by SEC were lower than the block copolymers’ global molecular weights, calculated from the PEG and PMLABe molecular weights, resulting from the fact that THF is not the best solvent for the block copolymers and that the measured molecular weights are related to polystyrene standards having a different macromolecular structure than the one of PMLA derivatives. However, SEC analysis allows determining the molecular weight distribution (Ip = Mw/Mn) and verifying the obtaining of a block copolymer (only one peak). In our study, SEC chromatograms of both block-copolymers highlighted the presence of only one macromolecular species (only one peak) and a good control of the polymerization reaction, as shown by the quite low polymolecularity of the samples (Ip < 1.5) in light of these results, we were able to obtain well-defined block copolymers suitable to continue the study of NPs’ preparation. However, we still have to further characterize the block copolymers, especially to determine their thermal properties (Tg, Tm) by differential scanning calorimetry (DSC), which gives an insight on the copolymer state (rubber or glassy state). Nevertheless, we can have a primary idea of the block copolymer’s thermal properties since it should have both the thermal properties of each block, i.e., the ones of PEG (Tg = −20°C, Tm = 50°C) and the ones of PMLABe (Tg = 37°C).

**Table 1:** **Characteristics of the synthesized block-copolymers**.

Block copolymers	M_PEG_^a^ g/mol	M_PMLABe_^b^ g/mol	Mw^c^ g/mol	Ip^c^
PEG_42_-*b*-PMLABe_73_	2 015	15 860	5 430	1.38
Biot-PEG_66_-*b*-PMLABe_73_	3 274	14 630	5 510	1.45

*^a^Given by the supplier (PEG IrisBiotech)*.

*^b^Determined by 1H NMR using the relative intensity of protons of PEG and PMLABe blocks*.

*^c^Measured by size exclusion chromatography (SEC) in THF at 40°C with poly(styrene) standards (1 mL/min, RI detector)*.

Nanoparticles can be easily and reproducibly prepared from these amphiphilic block copolymers by the nanoprecipitation technique, consisting in a rapid addition of a water miscible solvent, in which the block copolymer was dissolved, to an aqueous solution under vigorous stirring at 20°C (14–16).

For the standard method, acetone was eliminated under vacuum, while for the gel filtration method, empty NPs and ^185/187^Re-SSS loaded NPs were passed through a PD-10 Sephadex G-25 Medium column. The prepared NPs were analyzed by: (i) DLS, giving access to the average Dh and polydispersity (Đ), (ii) Zetametry, giving access to the surface charge of the NPs, and by (iii) TEM, allowing determining the NPs’ morphology.

As shown by results gathered in Table 2, both the standard nanoprecipitation method with acetone and the gel filtration method (with DMF) allowed preparing both stealth and targeted NPs with Dhs varying between 65 and 99 nm with a quite small size distribution (Đ lower than 0.15), highlighting the formation of well-defined nano-objects in good agreement with the size of micelle-liked NPs (45). On the other hand, the gel filtration method with acetone led to larger NPs with a higher dispersity (Đ around 0.2). However, the values stayed acceptable. The radiolabeling procedure was first tested with the non-radioactive ^185/187^Re-SSS complex. The preparation of ^185/187^Re-SSS loaded NPs by the standard nanoprecipitation method with acetone led to the formation of large aggregates with quite large size distribution (data not shown) and was not further used in this study. Therefore, we used the gel filtration method to load the ^185/187^Re-SSS complex inside the NPs. As shown by results given in Table 2, gel filtration with DMF allowed obtaining quite well-defined NPs (low Đ) with a slightly increased Dh, probably as a result of the presence of the complex inside de NPs. On the contrary, with acetone, it led to larger NPs with higher polydispersity indexes. At this stage, we can conclude that gel filtration with DMF seems to lead to the best results in terms of size and size distribution. Stirring the solution for 15 min, instead of 1 h leads to not as good results (Dhs and Đ, respectively, 144 nm vs. 104 and 0.19 vs. 0.14 for ^185/187^Re-SSS loaded PEG_42_-*b*-PMLABe_73_ in DMF), but it is better suited for the encapsulation of technetium-99m, due to radioactive decay considerations, and values are still satisfactory. The NPs prepared by the gel filtration methods (acetone and DMF) loaded or not with the ^185/187^Re-SSS complex have zeta potentials around −30 mV highlighting a negative surface charge which can be associated with a good stability of the corresponding NPs. Such negative surface charges can be the result of a block copolymer chain conformation allowing the presence of carboxylate end groups of the PMLABe block at the surface of the NPs thus lowering the values of the zeta potential. However, further experiments (static light scattering, Cryo-TEM, etc.) are needed to determine the morphology of the NPs and if any change can be observed in function of the preparation procedure used to obtain the NPs.

**Table 2:** **Characteristics of empty and ^185/187^Re-SSS loaded PMLABe derivatives-based NPs**.

NPs	Hydrodynamic diameter (nm)^a^	Polydispersity index (Đ)^a^	Zeta potential (mV)^b^
**PEG_42_-*b*-PMLABe_73_**			
Standard nanoprecipitation method with acetone	80	0.06	−6
Gel filtration with acetone	113	0.17	−31
Gel filtration with DMF	99	0.11	−29
**Biot-PEG_66_-*b*-PMLABe_73_**			
Standard nanoprecipitation method with acetone	65	0.12	−7
Standard radiolabeling method with acetone	80	0.21	–
Gel filtration with acetone	87	0.20	−26
Gel filtration with DMF	68	0.15	−30
**PEG_42_-*b*-PMLABe_73_[^185/187^Re-SSS]**			
Gel filtration with acetone	174	0.17	−37
Gel filtration with DMF	104	0.14	−32
**Biot-PEG_66_-*b*-PMLABe_73_[^185/187^Re-SSS]**			
Gel filtration with acetone	135	0.18	−25
Gel filtration with DMF	99	0.15	−39

*^a^Measured by dynamic light scattering (DLS)*.

*^b^Measured by zetametry*.

**Figure 2:**
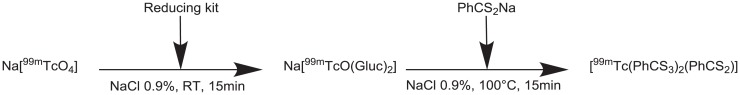
**Synthesis of the ^99m^T-SSS radiocomplex**.

In parallel, the selected radioactive complex [^99m^Tc(PhCS_3_)_2_(PhCS_2_)] (^99m^Tc-SSS) has been prepared in two steps, starting from the generator-eluted pertechnetate ion, [^99m^TcO_4_]^−^, in 0.9% saline solution (Figure 2) (44). ^99m^Tc-SSS complex was obtained with a RCP over 90%.

After those steps, we had to set up a simple and reproducible way of radiolabeling PEG_42_-*b*-PMLABe_73_ or Biot-PEG_66_-*b*-PMLABe_73_ based NPs. In a first attempt, we tried to encapsulate the ^99m^Tc-SSS complex in already formed NPs, previously obtained by the nanoprecipitation technique as described above. However, we were not able to encapsulate the complex inside the preformed NPs because of their destabilization by the reducing agent which is present in the kits used for the complex formation. Consequently, it was necessary to extract the complex in dichloromethane in order to eliminate the kit constituents. Dichloromethane was then evaporated and the radiotracer retaken in a water-miscible organic solvent, then added to the acetone or DMF solution containing PEG_42_-*b*-PMLABe_73_ or Biot-PEG_66_-*b*-PMLABe_73_ block copolymers.

To determine the encapsulation efficiency, we have measured the NPs’ radioactivity. For that, we have eliminated the non-encapsulated complex:

- either by centrifugation in the case of the standard nanoprecipitation using acetone: the supernatant was discarded and the pellet was again suspended into physiological serum. The total radioactivity of the flask before and after centrifugation and the radioactivity of the supernatant were measured. The encapsulation efficiency was given by the ratio between the radioactivity of the flask before centrifugation and the radioactivity of the flask after centrifugation and resuspension of NPs (Table 3). Some trials have been done with ethanol to solubilize ^99m^Tc-SSS after dichloromethane evaporation, but this solvent is more difficult to evaporate than acetone, so the latter one was preferred.
- or by gel filtration through a PD-10 Sephadex G-25 Medium column. The total radioactivity of the flask before and after Sephadex filtration were measured and allowed to calculate the encapsulation efficiency (ratio between the radioactivity of the flask before and after filtration) (Table 3).

**Table 3:** **Characteristics of radiolabeled PMLABe derivatives-based NPs**.

NPs	Encapsulation efficiency (%)^a^	Hydrodynamic diameter (nm)^b^	Polydispersity index (Đ)^b^	Zeta potential (mV)^b^
**PEG_42_-*b*-PMLABe_73_ [^99m^Tc-SSS]**				
Standard nanoprecipitation method with acetone	61	230	0.15	+0.2
Gel filtration with acetone	89	121	0.22	−37
Gel filtration with DMF	86	103	0.16	−38
**Biot-PEG_66_-*b*-PMLABe_73_ [^99m^Tc-SSS]**				
Standard nanoprecipitation method with acetone	82	160	0.23	−4.3
Gel filtration with acetone	87	118	0.16	−16
Gel filtration with DMF	89	249	0.22	−25

*^a^Determined by radioactivity measurement*.

*^b^Measured by DLS (Delsa Nano^®^ Beckman Coulter)*.

After the radioactivity decay, Dhs, polydispersity indexes, and zeta potentials of the complex-containing NPs were measured by DLS. As shown by results gathered in Table 3, ^99m^Tc-SSS complex was successfully encapsulated in both PEG_42_-*b*-PMLABe_73_ and Biot-PEG_66_-*b*-PMLABe_73_ based NPs, using standard nanoprecipitation technique, with an encapsulation efficiency varying between 60 and 80%. Non-PEGylated PMLABe based NPs were also investigated to encapsulate ^99m^Tc-SSS, but the resulting labeled NPs were not sufficiently stable in time to be studied after radioactivity decay. The radiolabeled NPs, obtained through the standard labeling method, present increased diameters with identical low polydispersity indexes, typically in the 0.15–0.23 range, and neutral zeta potentials in comparison to non-radiolabeled NPs (Table 3). Several reasons can explain the increase of the diameters measured by DLS: (i) a swelling of the inner-core by remaining acetone which is quite difficult to totally eliminate in the experimental conditions of the standard nanoprecipitation procedure used to prepare radiolabeled NPs, (ii) the presence of the radiotracer itself, or (iii) an aggregation of the NPs during the time necessary for radioactivity decay (3 days). Except for the simple PMLABe NPs, the NPs demonstrated good *in vitro* stability, with no size change over time (Table 4). Thus, the increase in size cannot be explained by the delay for the radioactivity to decay before measurements. The results obtained on empty NPs prepared using the same protocol than the one used for the preparation of radiolabeled NPs (standard radiolabeling method), and with NPs encapsulating non-radioactive complex based on “cold” ^185/187^Re evidenced that the presence of the complex leads to an increase of the NPs’ diameters without aggregation (Table 2). However, because the total elimination of acetone is quite difficult with the radioactive complex and centrifugation procedure is not satisfactory, with NPs still present in the supernatant, as evidenced by DLS measurements, thus probably minoring encapsulation yields, we tried to purify the radiolabeled NPs by dialysis. However, such method was time consuming, which is crippling with short half-life radionuclides, and led to no better results than centrifugation, so this method was not pursued.

**Table 4:** **Over-time stability of PMLABe derivatives-based NPs (measured by DLS)**.

NPs	Hydrodynamic diameter (nm)	Polydispersity index (Đ)
**PEG_42_-*b*-PMLABe_73_**		
D0 (acetone)	113	0.17
D7 (acetone)	119	0.13
D0 (DMF)	99	0.11
D7 (DMF)	105	0.10
**Biot-PEG_66_-*b*-PMLABe_73_**		
D0 (acetone)	87	0.20
D7 (acetone)	87	0.19
D0 (DMF)	68	0.15
D7 (DMF)	88	0.15

We therefore used the gel filtration method to prepare radiolabeled NPs. Using this gel filtration procedure, we also observed larger Dhs with slightly higher polydispersity index values compared to empty NPs (Table 3). This increase in diameters is probably the result of the radiotracer encapsulation, because no significant differences were observed in diameter and polydispersity index values between the standard nanoprecipitation procedure and the gel filtration method (Table 2). However, the encapsulation efficiencies seem to be slightly higher when gel filtration method is used either with acetone or DMF, with encapsulation yields between 86 and 89%. Moreover, this procedure is faster and easier to set up than the standard nanoprecipitation method because no centrifugation is necessary, thus avoiding the difficulties to separate the supernatant and the pellet. As observed previously with empty NPs, this method led to NPs with negative zeta potentials despite the PEG chains. As already mentioned, further experiments will be done to understand the reasons for this observed phenomenon which is probably related to the chain conformation adopted by the block copolymer as mentioned above.

**Figure 3:**
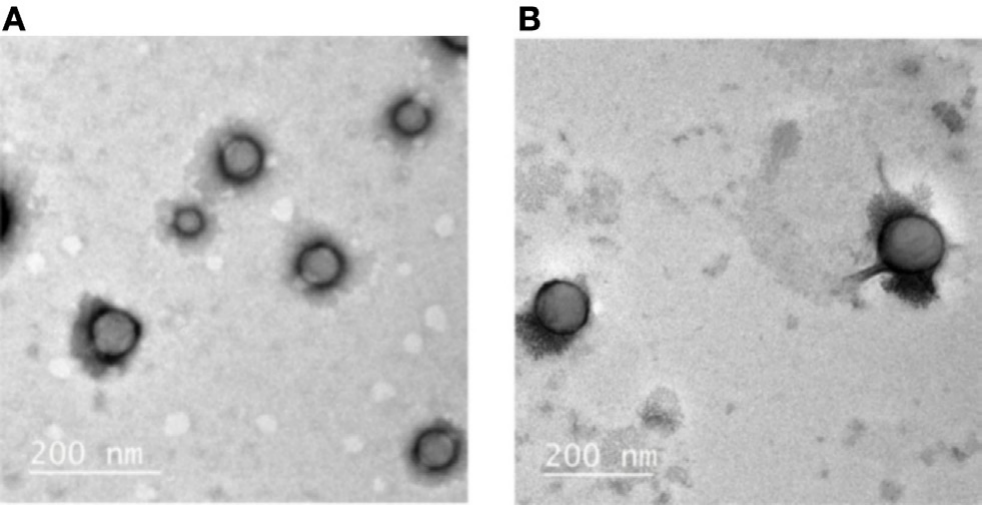
**TEM images of (A) Empty Biot-PEG_66_-*b*-PMLABe_73_ based NPs and (B) Radiolabeled Biot-PEG_66_-*b*-PMLABe_73_ based NPs**.

Both empty and radiolabeled NPs, after radioactivity decay, were analyzed by TEM in order to have an idea of their morphology (Figure 3).

Indeed, DLS allows only measuring Dh and size distribution without having access to NPs’ morphology. As shown by Figure 3, both empty and radiolabeled NPs, prepared with the standard method, have a spherical shape with a diameter in good agreement with the Dhs measured by DLS: around 80 nm for empty NPs and 120 nm for radiolabeled NPs. Moreover, an EDS analysis of the radiolabeled NPs was also realized during the TEM measurements: this technique allows the determination of the elements present in the analyzed sample (46). EDS analysis evidenced the presence of sulfur inside the NPs, reflecting the presence of the ^99m^Tc-SSS complex which contains sulfur atoms. On the contrary, EDS analysis realized on empty NPs did not highlight any presence of sulfur atoms. Therefore, it tends to prove that the ^99m^Tc-SSS complex is effectively encapsulated inside the NPs constituting by PMLA derivatives. TEM experiments now need to be done on NPs prepared using the alternative gel filtration method to get more insight on the influence of the preparation method on NPs morphology.

## Conclusion

Through this study, we highlighted that it is possible to obtain radiolabeled NPs based on PEGylated or biotinylated PEGylated degradable block copolymers with quite high encapsulation efficiency (up to 89%) in an easy and reproducible way. The radiolabeled NPs were characterized by Dhs ranging from 100 to 230 nm with quite low polydispersity indexes (under 0.2) and negative zeta potentials. The morphology of such NPs was shown to be spherical and the presence of sulfur, resulting from ^99m^Tc-SSS complex, was detected inside the NPs.

We are now moving to ^188^Re-SSS encapsulation and *in vitro* assays to confirm the potential of stealth and targeting NPs based on PMLABe derivatives as therapeutic nanovectors. Since our team has been working on DOX’s encapsulation and the grafting of a fluorescent probe too, we likewise envision to combine these different approaches as potential theranostic tools of interest for combined chemo-/radiotherapy and/or bimodal imaging probes for instance.

## Author Contributions

NL and SC-M designed and supervised the project, participated in the experiments and the analysis and interpretation of data, and wrote the manuscript. LC, MB, CB, CR, and MD made contribution in the design of the work, realized the experiments, and analyzed the results. NN designed and supervised the project and participated in the interpretation of data.

## Conflict of Interest Statement

The authors declare that the research was conducted in the absence of any commercial or financial relationships that could be construed as a potential conflict of interest.
